# Development of a Web-Based Resource for Parents of Young Children Newly Diagnosed With Autism: Participatory Research Design

**DOI:** 10.2196/15786

**Published:** 2020-09-30

**Authors:** Aspasia Stacey Rabba, Cheryl Dissanayake, Josephine Barbaro

**Affiliations:** 1 Olga Tennison Autism Research Centre La Trobe University Melbourne Australia; 2 Cooperative Research Centre for Living with Autism (Autism CRC) Brisbane Australia

**Keywords:** autism, diagnosis, parents, support, co-design, eHealth

## Abstract

**Background:**

The internet provides an ideal avenue to share information, advice, and support regarding autism. However, many websites lack quality control and rarely provide a one-stop resource for families to access necessary, evidence-based information.

**Objective:**

This study aims to use participatory action research (PAR) with end users (ie, parents) and clinicians to develop a web-based resource (Pathways Beyond Diagnosis) to improve timely access to quality, evidence-based information, and support for families after their child is diagnosed with autism.

**Methods:**

The PAR approach involves 4 phases: (1) cooperative researcher-stakeholder planning, (2) cooperative researcher-stakeholder–based action, (3) stakeholder observation, and (4) cooperative researcher-stakeholder reflection. A total of 15 participants (parents, n=3; clinicians, n=9; and researchers, n=3) attended individual or group participatory design workshops. This was followed by the translation of knowledge and ideas generated during the workshops to produce mockups of webpages and content, rapid prototyping, and one-on-one consultations with end users to assess the usability of the website developed.

**Results:**

A total of 3 participatory design workshops were held with the participants, each followed by a knowledge translation session. At the end of the PAR cycle, an alpha prototype of the website was built and a series of one-on-one end user consultation sessions were conducted. The PAR cycle revealed the importance of 6 key topic areas (understanding autism, accessing services, support, gaining funding, putting it all together, and looking into the future) associated with the time of diagnosis, which were incorporated into the beta version of the website.

**Conclusions:**

The development of the Pathways Beyond Diagnosis website using PAR ensures that families have ready access to practical and evidence-based information following a young child’s diagnosis. The website guides families to access relevant, reputable, and evidence-based information in addition to summarizing key challenges encountered after diagnosis (ie, grief, sharing the diagnosis) and the importance of self-care.

## Introduction

In the last 5 years, the proportion of users accessing the internet for health services or health research has increased two-fold from 22% in 2014 to 2015 to 46% in 2016 to 2017 [1]. For parents of children with autism spectrum disorder (ASD), the internet is the most frequently used approach to obtain information [2,3]. Unfortunately, the plethora of web-based information about ASD can be overwhelming and daunting for families [3]. Thus, knowing where to look and what to read is one of the greatest challenges in today’s web-based world.

When parents receive their child’s diagnosis of ASD, they are often left to navigate the world after the diagnosis on their own without much clarity on next steps. The receipt of a diagnosis often elicits strong emotional reactions from parents and can have detrimental effects on their stress, well-being, and quality of life [4-6]. As a result, it is important to ensure that families know where to turn for more information and support. Although the internet is the preferred medium for sourcing autism information, there are currently no guidelines for finding high-quality websites [7].

With ASD becoming increasingly prevalent, so is the amount of information available. In 1999, Charman [8] reported 104,950 results from a search of the term “autism,” and in 2009, the search by Reichow et al [7] (2012) produced 19,900,000 results. After 7 years, (July 4, 2019, 11:38 AM AEST), our search of “autism” returned 176,000,000 results using the Google search engine. This substantial increase in web-based information mirrors the increased awareness and interest in ASD over the last two decades, which also coheres with its increased prevalence [9] over this time. Such an increase makes it even more challenging for individuals, especially parents, to sieve through and determine useful, accurate, and reliable information to guide their journey with their newly diagnosed child. Existing research demonstrates that parents prefer to access information that can be tailored to the child’s and family’s specific needs [10]. Furthermore, parents rely heavily on local sources of information and value easier access to internet sources that they trust, indicating the need to provide customizable (and locally relevant) web-based information [11,12].

The availability of web-based information and support can enable families with immediate and free access to important and appropriate knowledge and services to assist them. However, web-based information can also confuse families, primarily owing to the sheer volume of information. It is therefore essential that parents are guided toward high-quality and reputable websites. To make this easier for families, our aim was to develop a web-based resource that provides useful postdiagnostic information about ASD and to guide parents to the appropriate web-based websites or services that may benefit them and their child. To develop this resource, we undertook a collaborative approach together with parents and clinicians to identify the necessary support for families soon after their young child’s diagnosis of ASD.

Previous studies [13-15] have identified that effective partnerships between researchers, service providers, and key stakeholders, in addition to encouraging individuals’ participation in research, can increase the quality of investigations and findings. Furthermore, there is a growing demand for neurotypical researchers to engage with the autistic community to increase the meaningfulness, applicability, and sustainability of research by taking into consideration the ethics, values, and beliefs held by the community [15].

Incorporating knowledge from the autistic community within a participatory action research (PAR) framework can be transformative for participants and researchers. PAR is based on 3 overarching principles: (1) reflection, (2) data collection, and (3) action. A unique method used to develop interventions with the direct input of stakeholders, PAR uses a research strategy aimed at improving health and reducing health inequities by involving people who, in turn, take action to improve their own health [16,17]. PAR seeks to empower individuals who are most affected by the research outcome and involves the collaboration of researchers with the population of interest to solve a problem or develop an intervention [16]. A key element of this process involves collective, self-reflective inquiry for both researchers and participants to understand and improve on practices in which they participate and situations in which they are engaged. PAR is linked to action, which ideally leads to communities having increased control over their lives [17]. This reflects and echoes the overarching principle of inclusion highlighted by the Disability Rights Movement: “nothing about us without us” [18]. Thus, PAR promotes a partnership model that is fundamental to participants holding equal power in research teams and enables local knowledge to be used to achieve relevant, appropriate, and high-quality end goals [17].

Previous studies have effectively used PAR to develop a range of web-based resources, including electronic learning and health promotion materials, an asthma self-management app, an electronic Individual Care Plan for children with disabilities, and a mental health e-Clinic [19-22]. All studies shared similar features where reflection, data collection, and action were used to co-design and build these resources with important insights and expectations brought to the forefront by end-user involvement. This research reinforces the benefits of end-user involvement in the development of web-based resources.

The rationale behind the use of PAR in the development of web-based resources about autism may indicate that active engagement of end users can reduce the 17-year gap that is common in translational research [23]. This paper reports the developmental process of a web-based resource for parents of newly diagnosed children with ASD. The aim of this study was to use PAR with end users (ie, parents of children with autism) and clinicians together with our research team to co-design and build a website to improve timely access and provide better guidance to evidence-based high-quality autism information and resources following a diagnosis of ASD within the Australian context.

### PAR

Participatory design methodologies, which were developed in the late 1960s and early 1970s, emphasize the importance of involving all stakeholders (including end users, developers, and researchers) in the design and development of resources to ensure that the end product meets everyone’s needs. It also improves usability and increases the engagement of users [19,21,24] with the end product. In collaboration with study participants and a professional advisory group, PAR was identified as the most appropriate participatory design methodology for the development of a website for families of newly diagnosed children with ASD.

The development process was based on the following steps: planning, action, observation, reflection, and using the new learning to inform further action planning [25,26]. Although PAR shares similarities with traditional research (ie, design, implementation, and evaluations), it differs in that the stakeholders are integral to all parts of the research process. Participants’ active role in the outcome sets PAR apart from traditional research methods where the participants tend to be more passive in their receipt of research outcomes [17]. A key component of PAR is flexibility; the observation and reflection steps promote cumulative learning, which enables modification to the outcome and encourages participant empowerment. Furthermore, PAR also enables participants to be realistic and specific about the practicality of the proposed intervention model.

### Objective

Our overall objective was to develop a web-based information model that empowers parents by providing them with timely access to high-quality evidence-based information and support following the diagnosis of their child with ASD. Using an informational model to improve parental and family outcomes, parents can be better prepared and more confident as they tackle their new journey in raising their child with autism. Furthermore, providing families with one key website that redirects them to other relevant high-quality resources can help them easily navigate the plethora of autism-specific information available on the (often confusing) World Wide Web.

We used the theoretical foundation of self-efficacy, which has been demonstrated to predict and promote behavior change and improve health outcomes in a range of conditions (eg, diabetes mellitus) [27]. Self-efficacy refers to an individual’s belief in their capabilities to organize and execute a course of action to produce given attainments [28]. Furthermore, the use of behavior change theory itself in intervention development has been associated with greater intervention effectiveness [27]. This, in combination with evidence-based content and strategies as well as key stakeholder input, serves to optimize individual and family outcomes.

In this study, data obtained from PAR informed the development of the abovementioned website for parents of young children newly diagnosed with ASD. Specifically, PAR was used to gather information about what parents need to know after receiving a child’s diagnosis and how to best provide this information within a web-based format to enhance accessibility and usability. The aim was to establish a *self-contained* website that does not require regular input by a health care provider or other third party. This information model can provide an efficient and economic avenue for health and government bodies and allow families to gain timely and ready access to relevant information to support their child and family’s well-being in the critical time after diagnosis.

## Methods

### Participants

The target population was recruited via convenience sampling through email invitation to autism industry partners and parents from a previous qualitative study [10]. As outlined in the PAR methodology, collaborative partnerships were formed between key stakeholders (ie, participants), including parents of children with autism, health professionals, and researchers. A total of 13 parents, who previously received their child’s early diagnosis of ASD (<36 months), were invited to provide knowledge and feedback regarding postdiagnostic support. Two mothers and one father (3/17, 23%) agreed to participate. Their demographic details are presented in Table 1. A total of 17 senior health professionals in the autism field were invited to participate in an advisory group for the development of this resource (Table 2). From these health professionals, 53% (9/17) agreed to participate, comprising 3 pediatricians, 3 psychologists, 1 psychiatrist, 1 social worker, and 1 occupational therapist.

 In partnership with autism researchers (n=3), the health professionals (n=9) and parents (n=3) comprised the overall participant sample of 15 individuals. As participants progressed through each PAR phase, some participant attrition was observed: Phase 1: n=15; Phase 2: n=10; Phase 3: n=9; and Phase 4: n=8, with an attrition rate of 46% from Phase 1 to 4. None of the participants received compensation for their contribution to this research.

**Table 1:** Parent participant profile.

Characteristics	Parent 1	Parent 2	Parent 3
Parenting role	Father	Mother	Mother
Age (years)	46	42	54
Marital status	Married	Married	Separated
Place of birth	Australia	Australia	Lebanon
Time elapsed since child’s diagnosis (years)	1.5	1	7
Child’s diagnosis	ASD^a^, LD^b^	ASD	AD^c^, ID^d^
Child’s age at diagnosis (months)	17	31	22
Gender of the child	Male	Female	Male
Household income, Aus $ (US $)	>175,000 (127,015)	95,000-115,000 (68,951-83,467)	35,001-55,000 (25,404-39,919)

^a^ASD: autism spectrum disorder.

^b^LD: language delay.

^c^AD: autism disorder.

^d^ID: intellectual disability.

**Table 2:** Professional participant profile.

Professions	Sex	Years of experience in autism	Specific area of expertise
Pediatrician	Male	15	Early diagnosis, comorbidities, teaching
Pediatrician	Female	28	Prevalence, cause, diagnosis, prognosis, intervention, research
Pediatrician	Female	38	Diagnosis, intervention, research
Psychologist	Male	22	Research
Psychologist	Male	12	Diagnosis, intervention
Psychologist	Female	10	Diagnosis, intervention
Psychiatrist	Female	20	Diagnosis, intervention, research
Social worker	Female	15	Child development, intervention, family well-being
Occupational therapist	Female	20	Intervention, diagnosis
Researcher	Female	35	Research in early diagnosis, early intervention, social cognition, parental experience, employment
Researcher	Female	14	Research in early identification and diagnosis
Researcher	Female	11	Diagnosis, intervention, family well-being, mental health, parenting, research

#### Phase 1

The initial participatory design workshop (n=15) was held with parents of children with autism (n=3), health professionals who work with families affected by autism (n=9), and researchers in the field of autism (n=3). The majority of these participants were female (11/15, 73%).

#### Phase 2

A total of 10 participants engaged in Phase 2, which consisted of parents (n=3), health professionals (n=4; 2 psychologists, 1 occupational therapist, and 1 pediatrician), and researchers (n=3), with the majority being female (80%).

#### Phase 3

A total of 9 participants remained involved in Phase 3, the observation phase, where a tentative website structure was developed (ie, alpha prototype). A group meeting of health professionals and researchers was then held (3 researchers, 2 psychologists, and 1 occupational therapist).

#### Phase 4

A total of 8 individuals participated in Phase 4 (one-on-one end user consultation sessions), who were predominantly female (7/8, 88%), comprising 2 mothers, 1 father, 1 psychologist, 1 occupational therapist, and 3 researchers.

### Procedure

Ethics approval was obtained from the University Human Ethics Committee. The meetings or workshops were conducted either face-to-face or virtually at La Trobe University in Melbourne, Australia in a secure, private room to ensure confidentiality. Participants who engaged virtually were advised to remain in a quiet and confidential space for the duration of the meeting. Given the plethora of autism information available on the web, it was important that this resource be optimized within the current web-based arena to ensure easy accessibility by parents, given all other available websites. On the basis of the literature on search engine optimization, there are several key features distinguishing good versus poor website optimization: (1) use of key phrases, (2) consistent web theme, (3) organized content, (4) optimized links, (5) professional communicators (ie, practitioners or researchers), and (6) web-based promotion through various reputable sources [29,30]. A qualitative research design using the PAR cycle guided the development of an informational model for parents after diagnosis. Features of website optimization were encountered, reflected upon, and discussed throughout the phases of development.

**Figure 1:**
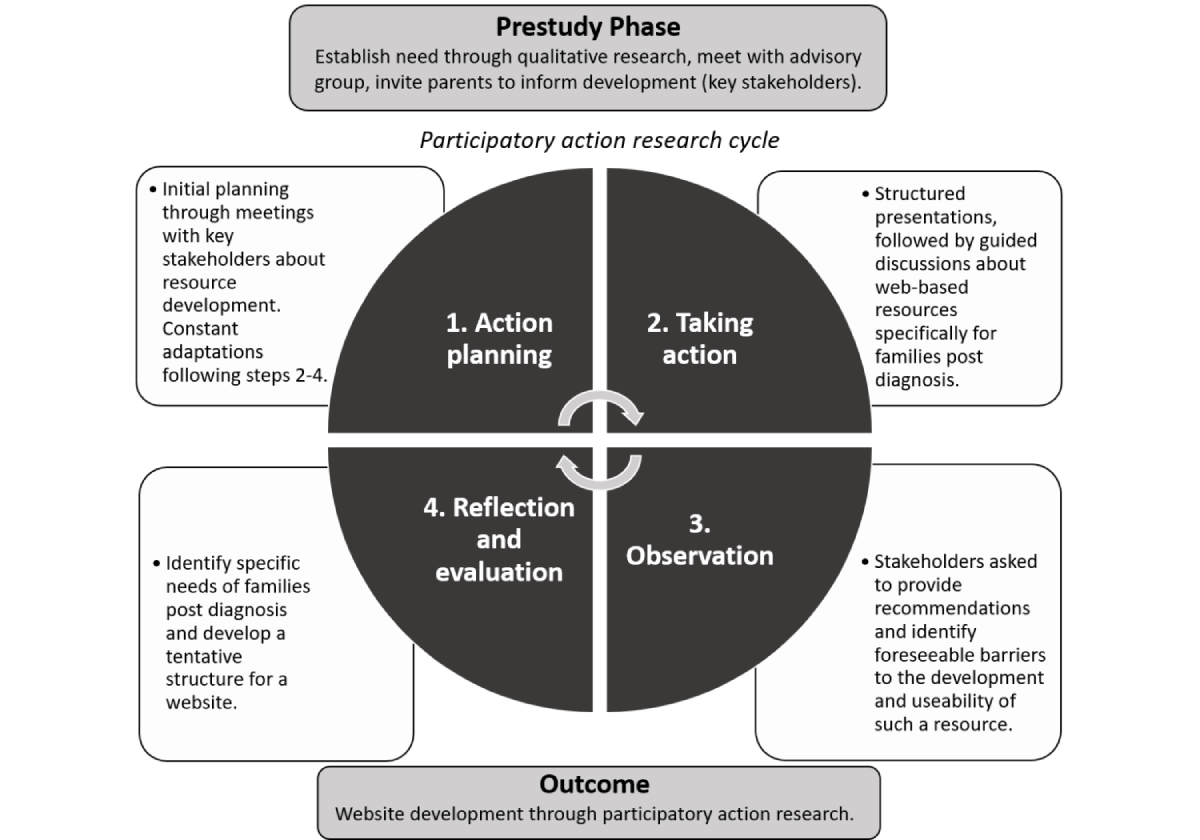
Model of participatory action research.

The PAR cycle involves 4 major phases: (1) action planning, (2) taking action, (3) observation, and (4) reflection and evaluation. These phases are depicted within the PAR model in Figure 1 and have been adapted from previous research by Munns and colleagues [31]. In this study, Phases 1 to 3 included participatory design workshops, where participants attended individual and/or group meetings. This was followed by knowledge translation sessions where cooperative researcher-stakeholder ideas generated during workshops were used to produce mockups of webpages and content. Finally, in Phase 4, rapid prototyping of the end product occurred and one-on-one consultations with end users assessed and informed the usability of the website.

A total of 4 participatory design workshops, 4 knowledge translation sessions and 3 one-on-one end user consultations were conducted over a period of 6 months. Throughout Phases 1 to 3, health professionals predominantly chose to participate in group meetings, whereas parents preferred individual meetings with the lead author or researcher.

### Action Planning (Phase 1)

A total of 4 participatory design workshops (1 group meeting for health professionals and 3 individual meetings for parents) were conducted as part of the *Action Planning* stage, each lasting 30 to 60 min. During Phase 1, researchers presented examples of current web-based information about autism, how it was being utilized by families, and where the gaps were regarding postdiagnostic support. Through consultation and cooperative researcher-stakeholder planning, ideas were generated for the development of a postdiagnostic resource for families of newly diagnosed children.

### Taking Action (Phase 2)

The knowledge and ideas generated in Phase 1 were translated into mockups of webpages. A total of 4 knowledge translation sessions (1 group meeting and 3 individual meetings) were conducted where structured presentations were given to stakeholders (health professionals and parents). Following the structured presentations, participants engaged in a guided discussion about what type of web-based resource would be beneficial and how it could be utilized to meet a family’s needs after diagnosis. Following these guided discussions, participants were asked to respond to 2 open-ended questions or requests: “Please list any recommendations you have for the development and dissemination of this online resource for parents” and “Please list any barriers you foresee in the development and use of such a resource.”

### Observation (Phase 3)

Qualitative results were compiled for the observation phase (Phase 3) and presented to all participants, including parents, health professionals, and researchers. A final meeting was held with the health professionals and researchers where the key stakeholders collaborated to develop a tentative structure for a website utilizing evidence-based information. In addition, the 3 parents also participated in individual meetings, with the lead author providing recommendations and identifying foreseeable barriers to the development and usability of this resource. On the basis of these results and through collaboration with the researchers, a plan was formulated by all participants to guide the alpha build of the website. On observation, participants emphasized the need for adaptations to this, with specific content added to accommodate parents of newly diagnosed children (ie, various topics added: grief or loss, family support, and funding information).

### Reflection and Evaluation (Phase 4)

Participants were provided with a hard and/or electronic copy of the beta version of the website for reflection and evaluation. Stakeholders were asked to reflect and evaluate the layout, contents, and utilization of the resource. One-on-one consultations were conducted with the 3 parents who also provided general written feedback detailing the pros and cons of the resource, together with suggestions for improvement. This participatory data collection strategy is described as beneficiary assessment (BA) and involves asking participants, specifically those who will be the intended beneficiaries, about their use of a resource, satisfaction with that resource, and ideas for changes that might be needed [32]. The BA process enables researchers to gain insights from the beneficiaries to improve the quality of development of an intervention or resource.

### Data Analysis

To address the specific research objective, an inductive approach to content analysis was adopted as an efficient process to categorize raw data and generate an information model. A moderator guide, including a series of questions, was used and revised during the research process [33] to guide discussion through each phase and development of the resource. Data from the participatory design workshops, knowledge translation sessions, and one-on-one consultations were collected. Examples of questions in the moderator guide are presented in Textbox 1. Prompts related to participant responses were used to guide the ensuing discussion.

**Examples of questions in the moderator guide.**

- What gaps do you see in post-diagnostic support for families of children diagnosed with autism?
- What questions do you receive from parents immediately after diagnosing their child?
- In retrospect, what would have been most valuable to you after receiving your child’s diagnosis?
- What barriers do you foresee in the development and use of a post-diagnostic resource?
- What do you consider essential information for families post diagnosis?
- How do you see this resource standing apart from what already exists on the web?

Thematic analysis [34] was used to interpret the qualitative data according to the following key themes: (1) website content, (2) user interface, (3) referral to existing resources, and (4) interaction of the website with stakeholders or end users. Data were thematically analyzed through categorization, clustering, and identification of the common subthemes that emerged through phases 1 to 4 [34-36]. Records of all information including meeting discussions, written feedback, and comments obtained in phases 1 to 4 were then grouped and interpreted by the lead author. Individual themes and related content were discussed by the researchers, rationale was given for grouping and interpretation of data, and differences in opinion were discussed until consensus was reached. A codebook was developed following data collection to guide the thematic analysis. The codebook was referenced throughout the data analysis to maintain consistency. Mechanisms such as peer debriefings and member checks were used for credibility, along with an acknowledgment of known research limitations to ensure trustworthiness of the data [37]. Information to be contained within this resource was evaluated based on 5 guiding principles (Textbox 2), which were used to determine the inclusion or exclusion of any links and key information within the proposed website model.

**Guidelines used to determine website inclusion.**

Standards for website inclusion:

- Peer-reviewed research literature (evidence based)
- Information developed by a tertiary institution
- Information developed by an academic or clinical expert in the autism field
- Details from a government organization
- Details from a leading not-for-profit autism organization

## Results

Iterative participatory design workshops, which formed the basis of the PAR cycle, were utilized for the co-design and building of this resource. The workshops revealed the importance of 8 key components that would be fundamental to the development of a user-friendly website:

1. A clear and succinct homepage.

2. An easy-to-read description of autism.

3. Information about how to access services to help the child.

4. Various supports that are available and links to existing websites or resources.

5. A summary of funding avenues.

6. A snapshot of practical strategies to help with child behavior.

7. A summary of how to make sense of the information—“putting it all together.”

8. A brief summary of information about the future.

**Figure 2:**
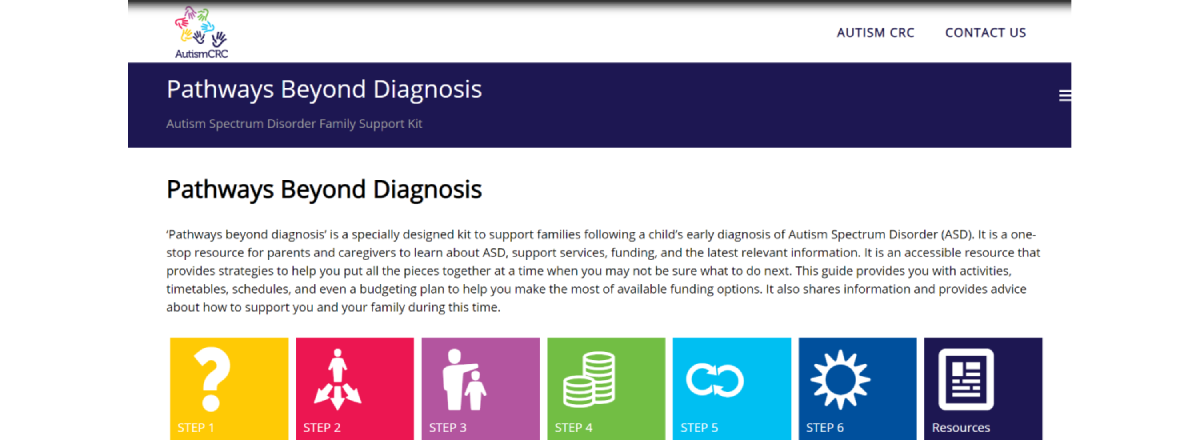
Component 1: Website Home Page.

**Figure 3:**
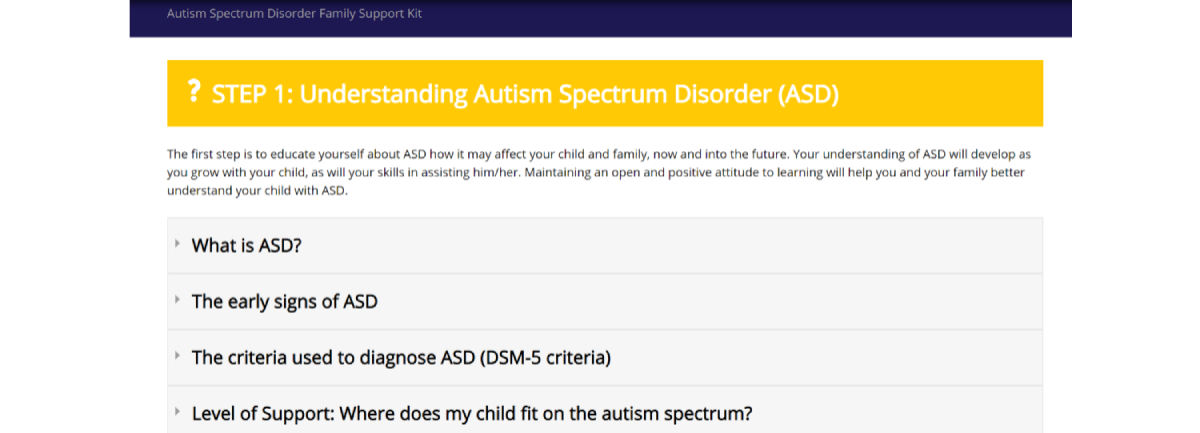
Component 2: Understanding Autism page.

**Figure 4:**
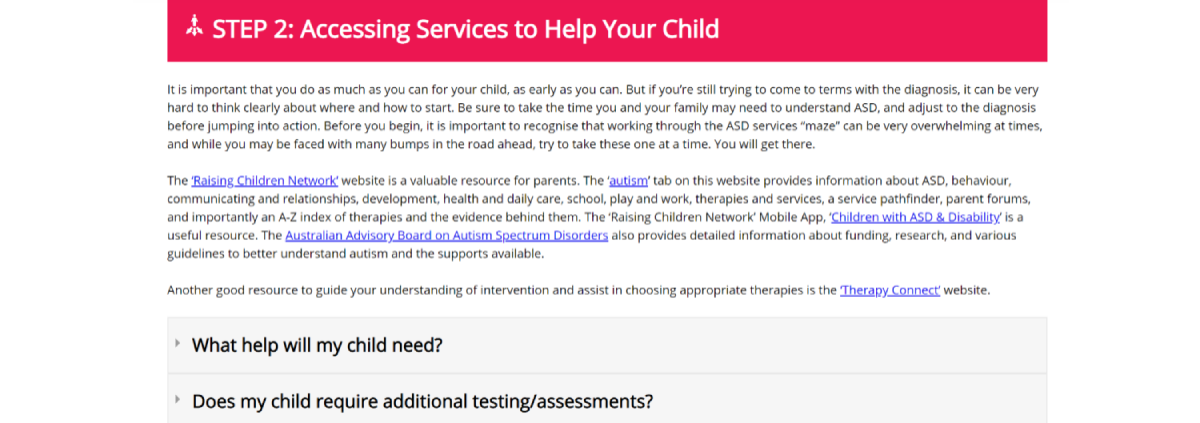
Component 3: Accessing Services to Help Your Child page.

**Figure 5:**
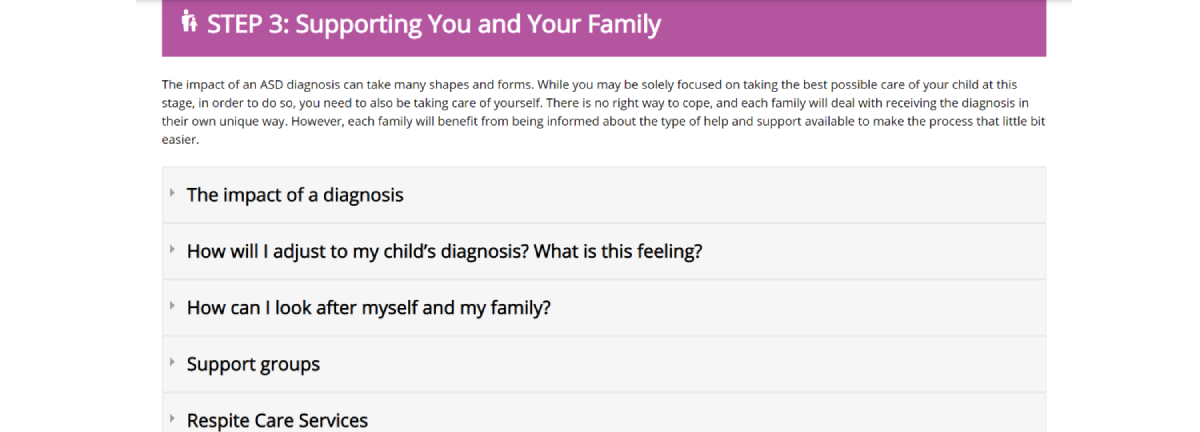
Component 4: Supporting You and Your Family page.

**Figure 6:**
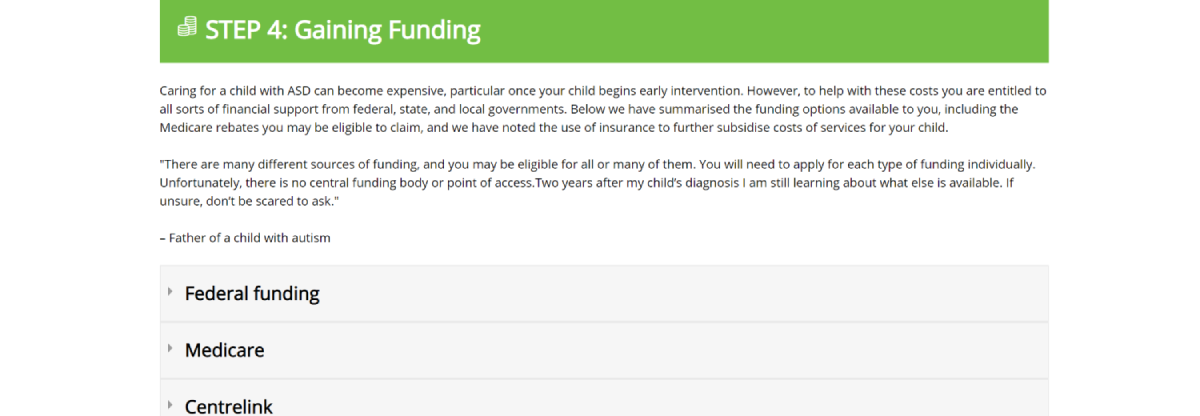
Component 5: Gaining Funding page.

**Figure 7:**
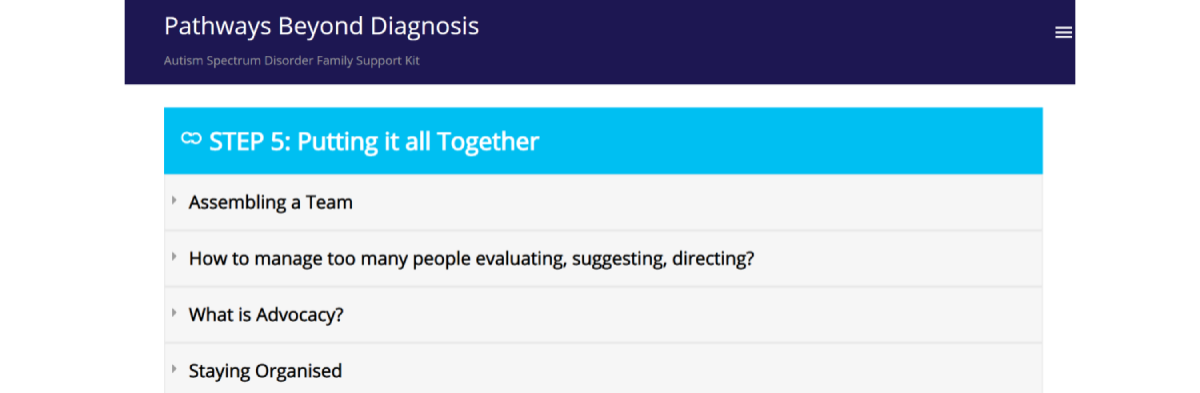
Component 6: Putting it all Together page.

**Figure 8:**
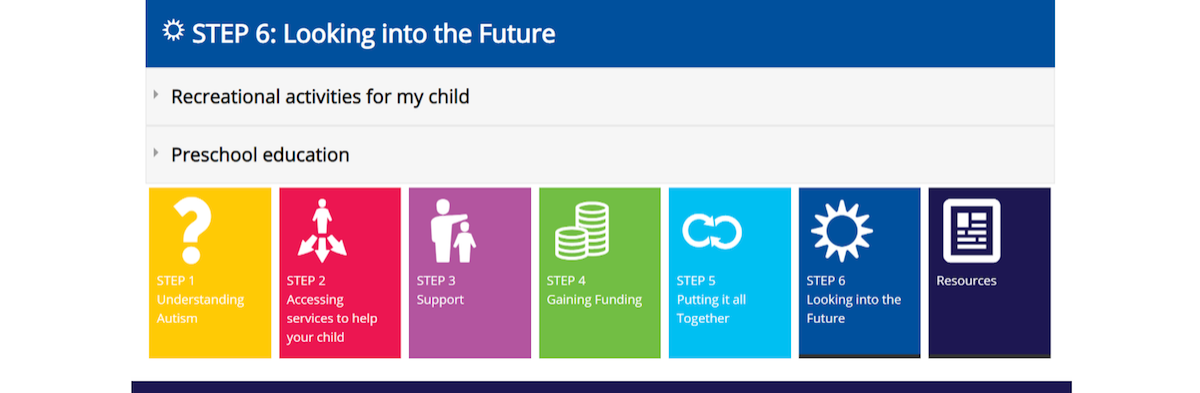
Component 7: Looking into the Future page.

These specific components (Figures 2-8) informed the development and the alpha build of the *Pathways Beyond Diagnosis* website. Each of these 8 components can be accessed directly, providing the parent or primary caregiver with the option to access the information most relevant to them and their family at the time. The entire site can also be accessed sequentially, if preferred.

### Component 1: Website Home Page

In relation to the user interface of the homepage, parents and health professionals agreed that it should be easy to navigate without using “too much text.” Thus, short and concise subheadings were used to direct individuals to the relevant content area. As most participants preferred icons instead of text, they were adopted as illustrated in Figure 2. Participants suggested the removal of a “contents style” page and lengthy instructions, opting instead to provide parents the opportunity to self-explore the website and navigate to the subtopics of their choice. Participants also proposed that “diagrams and tables” be used to describe ideas and summarize information instead of blocks of text to ease comprehension and reduce the common experience of parents “feeling overwhelmed” with information after diagnosis.

### Component 2: Understanding Autism

Participants suggested the use of realistic and practical examples when describing characteristics of autism, allowing those who access the resource to easily make sense of the definitions. In addition, participants noted that some of the language describing autism was not user friendly, describing it as “autism-expert speak.” Descriptions were therefore simplified using lay language to ensure clarity and comprehension, as participants emphasized that the reader, who is new to the autism world, “may not yet understand this” terminology, causing additional stress in trying to make sense of it.

### Component 3: Accessing Services to Help Your Child

All participants reported that there is "so much information available online," the website should be focused on redirecting parents to the "best information" that already exists. In addition, they recommended ensuring that parents of young children are educated on “selecting an intervention that is right for them.” Participants also stressed the importance of defining terminology and the careful use of language. Parents stated that “the term early intervention means absolutely nothing to someone outside the field, until they are familiar” and suggested that specific examples be given. They also suggested that the website note that “parents do not need to do all the interventions available to them,” but instead they “can try various approaches to see what their child responds to and suits the family best.”

### Component 4: Finding Support

*Finding support* was centered on parental self-care, adjustment to diagnosis, access to support groups, where to get help, and how a parent can help their child in their everyday interactions. Initially, the section on *finding support* focused heavily on parent support and self-care. However, parents expressed that although it was important to include information about parental self-care, following a diagnosis, a parent’s first focus is “What will help my child? How can I find support for them?” rather than on how to help themselves. Taking participant feedback into account, the section on *finding support* was restructured to include information for parents on how to support themselves, their family, *and* their child.

### Component 5: Gaining Funding

Given the different sources of funding provided by various bodies, participants noted that families need to be made aware of these sources and their eligibility for them. It was also emphasized that the application process may be challenging as parents may have to apply for each type of funding individually; thus, the information provided within the website should highlight the specific funding and support available, with help regarding how parents access it.

### Component 6: Putting It All Together

Participants emphasized the need for a component that brings all relevant information together. To move onto the next steps, families need to have the knowledge of how to assemble a team, stay organized, and advocate for their child. Although having a primary service provider or *key worker* as a central point of contact may be ideal, participants commented that this is unrealistic as it is often difficult to find the right person and one who also has the capacity to do this. Participants noted that it is much more likely that the “parents will be fulfilling this role.” Furthermore, they stated that it is important to communicate this reality to parents of newly diagnosed children to prepare them for various tasks, such as organizing appointments with therapists and managing intervention schedules. To assist with this, participants suggested an *example schedule* be included within the website that families can utilize as a template, as this visual representation is helpful to “maintain focus.”

Advocacy was also highlighted by participants as an important element of the postdiagnostic journey. Parents are in the best position to advocate for their child, but many never have had to take on this role in the past. As a parent of a newly diagnosed child, they are faced with the new reality of becoming an *autism advocate* overnight. Therefore, participants felt it was essential that the website provide families with guidance on how to become a successful and effective advocate.

### Component 7: Looking into the Future

Finally, in relation to future-oriented activities (such as preschool, playgroups, and other recreational activities), participants suggested that autism-specific examples and resources be provided to families “because often parents aren’t aware these services even exist.” Participants also emphasized that parents should be made aware of “inclusion support,” support that may help their child and family transition into intervention and other early childhood environments and further help them feel a sense of belonging in the chosen setting. Participants also expressed the importance of sharing the reality that there is inconsistency regarding support services, such that parental expectations can be managed, and emphasizing the need for their advocacy skills that will benefit them. For example, some families may receive inclusion support in some services (ie, preschool) but not in others (ie, child care). Participants were also warned from focusing too far into the future as this may hinder the parent’s ability to focus on the present.

## Discussion

### Principal Findings

As stated previously, the aim of PAR is to develop interventions that take into account the ideas and perceptions of those directly affected by the circumstances [38]. Importantly, the PAR process emphasizes the involvement of all stakeholders (ie, parents, health professionals, researchers) and acknowledges the key roles they play in the development of the end product to help ensure that it meets everyone’s needs, improves usability, and increases engagement of end users. This study specifically aimed to develop an information model that described the critical domains or topic areas that parents would benefit from following a child’s diagnosis of ASD. Together, the key stakeholders (ie, parents, health professionals, researchers) identified the critical domains that should be addressed in this information model. Following participant feedback, a web-based resource was suggested as the preferred delivery mode of postdiagnostic information.

The *Pathways Beyond Diagnosis* website aims to provide a one-stop resource for families of newly diagnosed young children, including a summary of important postdiagnostic information: autism education, services and support, funding, and practical strategies. It will also redirect families to other useful and reputable websites already available on the web. On the basis of the existing literature and participant feedback, 6 components were identified as the most pertinent for families after diagnosis. Although the key factors are documented in the literature and available on various websites [39-41], there is no single website that addresses them in one place. Having access to a web-based resource, such as *Pathways Beyond Diagnosis*, provides families and clinicians with a unique navigation or information model that addresses many of the initial questions raised after a child’s diagnosis of autism, either via the website itself or by redirecting individuals to other relevant sources.

As the internet continues to be the most frequently used method of accessing information and support by families of children with ASD, there is an increasing need for high-quality evidence-based websites [2,7]. By engaging with end users to drive the development of *Pathways Beyond Diagnosis*, we are addressing the needs of families and clinicians and engaging with the autistic community to increase the meaningfulness and applicability of the end product [15].

### Strengths

A key strength of developing the *Pathways Beyond Diagnosis* website is that information was gained from key stakeholders for a range of perspectives. Importantly, the parent perspective was combined with that of relevant clinicians to inform development. Furthermore, this resource will be freely available on the web as well as be accessible as a printable PDF document. This means that it does not discriminate based on families’ socioeconomic status or geographical location. Furthermore, it was intended as a one-stop resource such that families can promptly be redirected to appropriate websites rather than reinventing the wheel and repeating information that already exists elsewhere.

There is great value in the use of PAR methodology as it allows participants to provide in-depth descriptions of the issues raised by parents and health professionals when accessing web-based information about autism. In addition, participants also had the opportunity to provide realistic advice around the development of practical web-based resources to support parents. Furthermore, the funding and service pages are tailored to the geographical location but can easily be adapted to suit other areas, contexts, and cultures.

### Limitations

Engaging a larger sample of parents whose child had previously been diagnosed with autism proved to be a significant challenge. As a result, the data collected were explored qualitatively and each participant’s feedback was examined individually. It is important to note this as a limitation as additional data may have further informed program development and improvement. However, there is value in the lived experience that was communicated in the 3-parent descriptive responses. The lived experience gives credence to the useful and practical elements of the parent data. One parent revealed that their child was diagnosed 7 years before the study and, although the child was diagnosed early (<36 months), the time elapsed as diagnosis was significantly longer than the other 2 parents (12-18 months). Hence, this is a limitation whereby the information and knowledge shared by this parent about postdiagnostic support may differ from the other parents with more recent child diagnoses. However, on examination of this parent’s data, this was not the case.

A further limitation includes the language barrier, as the website has not been translated into other languages, limiting the population it can benefit to those who only understand English. In future versions, consideration should be given to culturally and linguistically diverse populations to allow for greater equity in accessing such web-based resources. For example, provision for a translating option on the homepage. An evaluation of the website by parents who have used it following the diagnosis of their child will be an important next step to determine its usefulness, accessibility, and impact on parental well-being.

Although the web-based information platform may prove to be beneficial for many, it is important to note that the absence of a built-in blog or forum for parents to interact was identified as a limitation by participants. This is an important element to consider for future versions, particularly given the strength identified in social support as a moderator of stress in parents of children with autism and the benefits of *networked empowerment*, the term used to describe how parents find other parents and facilitate each other’s access to resources and support [42,43]. When parents connect with other parents experiencing similar challenges, they discover new ways to promote advocacy, access support, and maintain their well-being. Furthermore, limited access to developers and researchers meant that data could only be analyzed by 1 researcher and then reviewed by a team of researchers. Although this approach allowed research insights to inform design and development, additional analysts would provide greater reliability in the findings. Future iterations of the website should aim to address some of these limitations by including a built-in blog that gives families the opportunity to share experiences, learn from each other, and access peer support. Including a team of developers and researchers from the outset to focus exclusively on data analytics may also strengthen future website development. Despite these limitations, given that this website development is pioneering work, it is important that the academic community understands the process behind its creation.

A good website should be an ever-changing platform. Websites offer the flexibility to update information to accommodate simultaneous changes in knowledge and services as they occur in the real world. With this in mind, limitations of this research should be viewed instead as new learning that will help enhance future iterations of the *Pathways to Diagnosis* website.

### Conclusions

Our web-based information model is based robustly on a theoretical foundation of self-efficacy, which has been demonstrated to predict and promote behavior change and improve health outcomes in a range of conditions [27]. In addition, evidence-based content and strategies, together with stakeholder input and usability testing through PAR methodology, resulted in an optimally designed website to improve access to relevant information for parents of newly diagnosed children with ASD and complement health care delivery, particularly during the stressful time after diagnosis.

Consumers’ increasing use of the internet for health information and ongoing revolutions in social media are strong indicators that consumers are primed to welcome a new era of technology where new knowledge is available at their fingertips in the comfort of their own home. However, the full potential of web-based interventions and information models is hindered by limited knowledge regarding their efficacy and effectiveness, high prevalence of usability errors, and high attrition rates [27]. We intend to address these limitations by assessing the efficacy and effectiveness of the *Pathways Beyond Diagnosis* website, identifying characteristics associated with website use and attrition, including whether it is helpful and easy to navigate, website optimization, and understanding the benefits or disadvantages associated with such a resource. A summative evaluation of the website will examine implementation through a usability study and evaluation surveys to determine the impact on family quality of life and parents’ self-efficacy, empowerment, and improved knowledge following wide access to this resource. With appropriate information and resources, parents can work toward understanding the diagnosis, coping with their own emotions, and adapting positively to maximize personal psychological and family outcomes.

## Abbreviations

ASD: autism spectrum disorder
BA: beneficiary assessment
PAR: participatory action research
